# Osteopathic health care in aged care facilities: The experience of practitioners in an emerging practice setting

**DOI:** 10.1111/ajag.13362

**Published:** 2024-08-19

**Authors:** Maria Amorim, Jade Bennett, David McGarry, Brydie Moore, Paul Orrock, Lochlan Whitton, Stephen Dullard

**Affiliations:** ^1^ Southern Cross University East Lismore New South Wales Australia

**Keywords:** allied health professions, health Care for the aged, osteopathic medicine

## Abstract

**Objectives:**

Residential aged care facilities (RACFs) are an emerging practice setting for osteopaths in Australia. This study explored the experiences of osteopaths working in Australian RACFs, reviewed current trends and challenges and considers future developments.

**Methods:**

Purposive and snowball sampling were used to recruit osteopaths with experience working in RACFs. This was a qualitative descriptive study derived from verbatim interview transcripts. Data were analysed using a six‐step thematic analysis.

**Results:**

Eight interviews were conducted during January 2023. Thematic analysis identified common experiences between participants. These included positive aspects of aged care, perceived challenges of working in aged care, exposure to age‐specific conditions, benefits of working in multidisciplinary teams and perceived gaps in university education curricula in relation to geriatric populations.

**Conclusions:**

Employment in RACFs may offer a satisfying employment experience for osteopaths, albeit hindered by policy and funding inadequacy. The respondents suggested enhancing pre‐ and postgraduate education to better prepare and encourage the profession to engage in this health service.

**Policy Impact**

The provision of osteopathic health care in residential aged care facilities is an emerging service. Osteopaths who have worked in these facilities report a generally positive work setting, albeit significantly challenged by policy and funding inadequacy. They suggest preregistration placements, inclusion of elective units and continuing education in aged care to better prepare the profession for residential aged care employment.

## 1 INTRODUCTION

Residential aged care facilities (RACFs) are an emerging practice setting for osteopaths in Australia, with surveys showing only four practitioners working in RACFs in 2017^1^ and 26 in 2019.^2^ With increased workforce demand in the sector^3^ and changes brought about by aged care reforms,^4^ information about how osteopathy contributes to aged care is needed to inform stakeholders, raise public awareness and ensure osteopathic services are properly utilised.

Osteopathy is a government‐regulated profession under the Osteopathy Board of Australia.^5^ Osteopaths require 4–5 years of university training and are equipped with multiple therapeutic skills, which include manual therapy, rehabilitation, pain education, exercise interventions and health promotion.^6^ Traditionally, osteopaths work from private practices; however, diverse capabilities^6^ allow osteopaths to work in a variety of settings, such as hospitals, RACFs, government agencies and education.^7^ The profession is estimated to manage an average of 3.9 million patients per year,^8^ including older patients (>65 years)^9^ and, according to the Medicare data, the number of older patients seeking osteopathic care is increasing. In 2015–2016, 52,461 (35%) of the patients managed by osteopaths under the Chronic Disease Management scheme were aged over 65 years, rising to 115,255 (39%) in 2022–2023.^10^ Osteopathy has been demonstrated to play a positive role in the treatment of the older populations' health, including reduction in symptoms, fall prevention, shorter hospital stays, decreased medication, improved quality of life and better stress management.^11^, ^12^, ^13^, ^14^, ^15^, ^16^

In Australia, allied health services including osteopathy are available as part of subsidised aged care.^17^ In the previous Aged Care Funding Instrument, allied health services were employed solely for pain management, under the ‘Pain Management Program’, which was limited to soft tissue massage and the use of technical equipment (such as electro‐therapeutic equipment) for complex cases.^18^ Under the recently introduced Australian National Aged Care Classification (AN‐ACC) system, services are tailored to an individual's needs and preferences based on their individual assessment.^17^ There are several implementation gaps in the new system, which compromise the use of allied health services,^19^ including a lack of designated funding and minimum minutes benchmark for allied health, resulting in a decrease of this type of care in RACFs.^20^ Residential aged care facilities are responsible for developing the care plan for each resident including allied health care,^17^, ^21^ and this decision is dependent on the providers' knowledge about these services.^19^

The literature on osteopathy's participation in the aged care sector is limited. Orrock^22^ reported on the percentage of osteopaths treating older patients, focusing on private practice. Adams et al.^8^ included data on osteopaths working in institutions, such as RACFs, but specifics on RACFs were lacking. Additionally, a paper in Osteo Life magazine^23^ featured potential benefits for older patients from osteopathy in RACFs, with insights from practitioners, but this had limited scholarly depth.

Qualitative data offer valuable insight into little researched fields.^24^ This study reports on the experiences of osteopaths working in RACFs, helping to explore aspects within osteopathy and aged care and identify potential avenues for further investigation. The results of this study may also inform relevant stakeholders, such as aged care providers and consumers, policymakers and the osteopathic profession about its role in RACFs.

## 2 METHODS

### 2.1 Aim

This study aimed to explore the experiences of Australian osteopaths providing health care to residents in RACFs.

### 2.2 Study design

This was a qualitative descriptive study based on individual interviews. The qualitative data were derived from verbatim interview transcripts. The COREQ checklist was used to provide methodological rigour.

### 2.3 Participants

Inclusion criterion was registered osteopaths who had experience working in RACFs in Australia, and practitioners were excluded if they were unable to be scheduled for an interview during the data collection period.

### 2.4 Recruitment and data collection

Purposive sampling was used to initially recruit participants, with the addition of snowball sampling to identify other participants. Eleven potential participants who were known to practice in RACFs were contacted via publicly available practice phone numbers and then provided with a participant information sheet and consent form by email. They were asked to forward the request to any other potential participants, which led to one additional participant.

### 2.5 Data collection

Interview questions were developed by the research team (Table 1), and semistructured interviews were conducted remotely on video (Zoom Inc.) by one interviewer (Author 2) and moderated by another team member (Author 4 or Author 6) to ensure consistency. The primary data collector (Author 2) and moderators (Authors 4 and 6) were osteopathy clinical masters' students who received interview training and practice from members of the team who are experienced postdoctoral researchers. The 30–40‐min interviews were conducted privately to ensure confidentiality, and participants were de‐identified in all data with a numerical code. All participants knew that the researchers were osteopathic masters' students and academics at the university, and none of the researchers had private relationships with the participants.

**TABLE 1:** Interview questions.

1. Could you briefly outline your experience as an osteopath working in an aged care facility?
2. How did you get involved?
a. How long after graduation did you start?
b. What was your level of experience when you first started? How many years in practice?
3. What is your role in the aged care facility/ies you work in? Can you be specific about the name of your role?
4. Could you please describe what you do in that role? Do you use manual therapy/ osteopathy?
5. In your experience, how does working in an aged care facility differs from private practice?
a. What are the challenges and rewards? What is your level of satisfaction?
b. What osteopathic skills are you employing and what are you not employing?
6. What advice do you have for osteopaths who are entering this field?
7. What do you think are beneficial skills to have specifically for an aged care setting?
a. Is there anything you wish you had known before entering the workplace?
b. Is there any specific equipment needed?
c. Do you need to provide this or is it provided by the facility?
d. Would you recommend the university to incorporate any of these additional skills into the curriculum?
8. Is there, or was there anything missing from your training?
a. What skills do you think would be needed?
9. Did you undergo any extra training for this job?
a. What was it?
b. Was it part of the job training, or did you seek it independently?
10. Is there anything else we have not asked that you would like to add?

Interviews were conducted once during January 2023, and closed caption functionality was used to generate transcripts. The interviewer and moderator cleaned and cross‐referenced all transcripts immediately after the interview to ensure the interviews were transcribed verbatim, and there were no mistaken words.

The Human Research Ethics Committee of Southern Cross University approved the study (Approval Number: 2022/013).

### 2.6 Data analysis

Data were analysed from audio transcripts using a six‐step thematic analysis.^25^ The initial coding of the transcripts was completed by the interviewing and moderating researchers (Authors 2 and 4), then two other research team members reviewed the coding on two transcripts to confirm their authenticity. These data were examined for various meaning units, which were then condensed and defined as specific subthemes. Categories were created and grouped, from which themes were created to link underlying meanings between each category. The thematic analysis was a continual process and took two rounds of refining before the final themes were identified, with direct quotes used as supportive evidence for each theme.

## 3 RESULTS

### 3.1 Participants

Eight osteopaths participated in the interviews, the other four were not available during the data collection times. No further interviews were conducted as researchers agreed that there was sufficient overlap of responses. All but one of the participants were male, with variable length of experience in osteopathic practice. Demographic data are outlined in Table 2.

**TABLE 2:** Demographic characteristics of interview participants.

Characteristics	Sample (*n*)
Age	
25–35 years	6
36–45 years	1
46–55 years	1
Gender	
Male	7
Female	1
Employment status	
Full time	1
Part time	7
Education level	
Bachelor's degree	2
Master's degree	6
Years of experience	
Clinical practice	
<1 year	1
1–2 years	3
3 + years	4
Aged care facility	
<1 year	1
1–2 years	6
3+ years	1

### 3.2 Thematic analysis

Five themes were constructed from the participant responses: positive aspects of working in aged care, challenges associated with working in aged care, perceived gaps in current university curricula and osteopathic techniques used by osteopaths in aged care.

#### 3.2.1 Positive aspects of working in aged care

All participants articulated positive aspects of working in aged care. Financial security was discussed within two contexts: that of a recent graduate and that of working during the height of the COVID‐19 pandemic:

Say you're a subcontractor moving to a new area or… you are just starting up your business, having some guaranteed income is anxiety‐reducing…I went into private practice (PP) right at the start of COVID. So, that probably played somewhat of a catalyst in terms of my moving into an aged care role in addition to having a private practice role… my primary practice was a gym environment which closed for a long enough period of time. (P1)

The variety of experiences associated with aged care work was another positive theme in these data and was described as less demanding when compared to PP:

I find it a lot less mentally taxing working aged care because I'm not having to constantly be thinking red flags, because they have doctors for that. (P7)

Most participants described the positive aspect of using aged care work as a supplement and balance to PP:

I came about that job just looking for something a little bit different, outside of private practice. (P7)

Exposure to chronic and advanced conditions that would not usually be seen in PP, and gaining experience working with these conditions was seen to be invaluable for the participants:

The range of chronic conditions that you'll see in aged care is completely different to anything you'll see in private practice. (P6)

You get to work with people towards the end stages of life. You need to apply different perspective to your treatment and your therapy and what you're trying to accomplish, what's realistic for people, what's actually important for them. (P2)

Participants identified numerous benefits for the residents as a direct result of their interventions:

They do a mobility review and then maybe do it in three months' time…their mobility's improved out of sight. It's good. (P6)

Improving biopsychosocial aspects among residents through social contact was also identified as a positive factor:

… the staff and the facility carers very much become family… there is an element of satisfaction that you do get out of giving a service to people that otherwise might not have a (social) interaction like that. (P1)

Participants noted that they often worked closely with several other health‐care professionals in a multidisciplinary team:

I've found it really rewarding because we work as a team regardless of the background of your studies. (P8)

… when you're working with carers and nurses and GPs that are in a facility, we have to communicate through case managers and nurses …. will give you some exposure in terms of communicating across a healthcare landscape. (P1)

Additionally, this interdisciplinary co‐operation allows for interprofessional learning, which participants found to be valuable for their continuing development:

Good way to meet physios and OTs and chiros as well… you can share and talk and learn about different modalities and you get to practice things… so it was very useful in terms of training and teaching. (P2)

I'm very lucky I've got senior physios, who I work alongside, and one of them does primarily hospital work and the assessments for the facility. So being able to learn from them has been fantastic. Yeah, and just bounce some ideas of what they would do with their lens and what we might do with our lens. (P1)

#### 3.2.2 Challenges associated with working within the aged care system

Throughout the interviews, participants noted various issues and negative aspects of this work, with participants emotionally impacted by the experience:

… sometimes it can be… emotionally draining because you're seeing a lot of these people sort of end of life. (P7)

People pass away regularly…it can become a hard job at times and particularly when you have runs of people who decline quickly… We see people who are otherwise fully functioning die in short timeframes. That's definitely challenging in terms of practitioner burden. (P1)

Participants identified lack of staffing as a ubiquitous issue on staff morale, and that also stretched scopes of practice:

… it's very, very short‐staffed. (P8)

You're forever being asked to help the staff… it's not your role… and actually you shouldn't do it, because if you do try to care for somebody and you do make a mistake, you're, you know, you're out of your scope of practice. (P2)

Low job satisfaction was a commonly mentioned, with the tasks assigned to the job description seen as unfulfilling:

Not necessarily a job that a full‐time position offers a lot of job satisfaction. (P5)

Very boring. However, there were ways of making it more osteopathic and clinically interesting. (P2)

Various challenges regarding treatment were noted:

I think some of the challenges, certainly the cognitive deficits in the residents, where the residents are not able to comply with what you want them to do. (P3)

Sometimes you wouldn't find them, sometimes they'd be in hospital, sometimes their family will be visiting, and you'd have to go back 2,3,4 times in the day to find them. (P2)

Feeling ill‐prepared and lacking training before the commencement of work were other challenges commonly identified throughout the interviews:

The first company I worked for did actually provide hands‐on training on the day, but it wasn't very good. It was more, show me around the facility, off you go, see you later. (P2)

… kind of would have liked a little bit more preparation for it, particularly when it comes to those with cognitive decline or poorer cognitive functioning. (P1)

#### 3.2.3 Gaps in curriculum

Participants noted areas in which they felt osteopathic courses could be changed to encompass the emerging role of osteopaths within the Australian aged care system, for example, neurodegenerative diseases:

I wish I had, a little bit more knowledge and understanding of the progression of the different types of dementia and how that can manifest because it's sort of like you have a vague idea. (P7)

… Little things about how to treat… how to properly perform the assessments, how to properly speak to people who have a cognitive deficit. (P3)

Some participants noted that while this information may help prepare graduates for aged care work, it may not be feasible if postgraduate osteopaths were unlikely to pursue a career in aged care work:

Would it (extra training) be helpful if you end up in aged care? Sure. Would it be helpful with anything in private practice? No. So it depends what you're producing the product out of the university for. (P1)

Participants felt their current education had not specifically focused on aged care, rather it centred around PP:

All of our training was for private practice. (P6)

I would have thought through some sort of… hospital training… that the other professions have is that, the transfers and mobility in bed, and from sitting to standing and ambulating. (P8)

Some participants noted that introducing electives to the university curriculum could be beneficial:

You'd almost have it as like a fourth or fifth‐year electives where someone that was interested in that could do that. (P5)

Participants also highlighted the importance of student placements in aged care facilities for gaining real‐world experience, that other professions have:

Student placements. The physios and OTs can have student placements at aged care… the right sort of student placement for osteopaths would also help with understanding oh, the health system is bigger than just private practice. (P8)

#### 3.2.4 Techniques used in the aged care setting

Working under the Pain Management Plan, health‐care professionals can only use therapeutic massage as a treatment modality. This rigidity in treatment intervention was a point of controversy:

[inflexibility of modality] was a point of contention amongst some of the companies and practitioners because a lot of the practitioners didn't have a background in massage. (P2)

It was often noted that participants would use other modalities outside the PMP‐specified techniques to attain results with the residents, where technique selection was based, to an extent, on resident preference:

Sometimes they didn't want their shoulder worked on four times a week… if you have their consent and it was reasonable, you could work on other areas… I would often do some exercises with them or do something active if they did not want hands‐on therapy in that area. (P2)

Some participants also noted that passive therapies such as articulation and massage were the best fit for certain residents due to their cognitive ability to coordinate active movement or follow instructions:

Yeah, I use a lot of counter‐strain. I use a lot of articulation. Occasionally I might use MET (muscle energy technique) depending on how responsive… how cognitive I should say the resident is. If they're able to respond to me and interact with me appropriately. (P3)

## 4 DISCUSSION

This study identified several themes of the experience of osteopaths working in RACFs. Parallel to the satisfaction of helping to improve residents' well‐being and functional capacity, participants felt their personal relationship with residents was an important and rewarding aspect of their work. They recognised that, in the absence of family and relatives, their interactions also fulfilled a social role, helping to maintain the mental and emotional well‐being of residents. This is in accordance with previous studies, where allied health professionals^26^, ^27^ RACFs management^28^ and consumers^29^ viewed the personal relationship between staff and residents as an important aspect of aged care, but expressed concern about the recent reforms, in particular, a concern that the pressure of meeting personal care minutes benchmarks tends to foster time focused care at the expense of quality personal interactions.^28^ The variety of professional experiences and diverse employment possibilities offered by aged care work are findings that could prevent attrition from the profession, in accordance with Fitzgerald and Vaughan,^30^ and supporting the discussion by Mastronardo et al.^31^ that increasing professional workforce readiness for more diverse employment settings may foster job satisfaction and sustainability for the profession.

Relationships developed through the multidisciplinary workforce in RACFs positively impacted participants' experience by enhancing their clinical knowledge and interdisciplinary care proficiency. Interprofessional collaboration is an important aspect of health care,^32^ a specific capability expected of osteopaths,^6^ and has been reported in Australian osteopaths.^22^ Vaughan et al.,^33^ however, identified that osteopathic students needed to increase their awareness about the importance of interprofessional co‐operation, and to consider how osteopathy is viewed by other professions. Mastronado et al.^31^ also identified the need for Australian osteopathic curricula to introduce teamwork development strategies to better prepare future clinicians for diverse work opportunities, for example by team‐based projects and clinical placements.

All participants identified insufficient funding and staffing levels as negative aspects of working in RACFs. These issues were highlighted by the royal commission into aged care^34^ and other studies,^3^, ^35^ and identified by several others as a source of low job satisfaction in RACFs^20^, ^26^, ^27^ and decreased quality of care.^20^, ^26^, ^27^, ^28^, ^29^ Although the Department is incentivising workforce development and increasing the budget allocation for aged care,^4^ recent financial evaluations^3^ indicate the cost of aged care provision is increasing, and the current budget may struggle to meet the mandatory direct care minutes, particularly due to workforce paucity and wage inflation. Further research is required to assess the cost and place of osteopathic health care in the context of RACFs.

Participants saw the limitations posed by their role under the pain management program as negative aspects of RACFs work. They recognised how osteopathic skills and techniques other than soft tissue massage could have been indicated and beneficial to RACFs residents. A similar experience has been reported by physiotherapists^26^ and occupational therapists^27^ working in RACFs, where the pain management program parameters prevented allied health practitioners from utilising their full scope of practice. The pain management program no longer exists under the AN‐ACC, and although the new system potentially allows for more flexibility,^17^ the question remains whether funding is sufficient and if effective strategies are in place to ensure individual care plans are developed with appropriate use of allied health.^19^ Preliminary data already suggest a significant decrease in allied health provision, where the prereforms'^8^ minutes per resident per day average has reportedly decreased to less than 5 min,^3^ falling short of the recommended ideal 22 min.^35^ Experts fear the detrimental consequences this could have on the well‐being of residents and the loss of allied health workforce as professionals leave the sector due to deteriorating working conditions.^3^, ^20^ This work has demonstrated that the satisfaction of osteopathic practitioners would likely be affected by limiting resident contact time any further. Participants suggested that preregistration curricula could include student placements in RACFs and electives focusing aged care. Following a review of national university curricula and input from an advisory board, Mastronardo et al.^31^ highlighted the need for changes in curricula to better prepare osteopaths for work in various contexts outside private practice. Pre‐graduate placement, core electives and postgraduate advanced practice programs were also identified as strategies to improve professional interest, preparedness and employability.^30^ Due to their lack of clinical exposure to specific and complex clinical presentations common in RACFs, participants initially felt unprepared for their new role. Abrey et al.^36^ ^(p.7)^ noted that ‘providing students with opportunities to experience a diverse range of case presentations is a challenge’. Clinical placements for students in RACFs could increase their exposure to the clinical environment and complex conditions typical to that population. These findings warrant consideration by Australian universities offering osteopathic courses as the profession endeavours to promote employment diversity. These data exploring an emerging health service for osteopaths in Australia suggest that it provides flexibility of employment, work satisfaction and may reduce attrition from the profession.

### 4.1 Limitations

This study was conducted in part by postgraduate students with limited experience in qualitative data collection and thematic analysis. To mitigate this, they undertook a preparation course for the interviews and collaborated with experienced researchers and supervisors throughout the project. Interviews were conducted before the AN‐ACC was introduced; therefore, these data could not reflect participant's experiences of recent changes brought by the new reforms.

## 5 CONCLUSIONS

Osteopaths working in RACFs report that their experiences are positive in terms of benefits to the residents, increasing the variety of practice, multidisciplinary learning and stable employment. The challenges relate to systemic funding issues and limits of scope, and they would be better prepared by pregraduate placements and core electives specific to aged care. Continued qualitative investigation into the experiences of osteopaths and other allied health practitioners within RACFs, reflecting the changes brought by the AN‐ACC, is imperative. Moreover, ongoing research to evaluate the impact of these changes on the utilisation of allied health services, including the quantity and appropriateness of allocation within residents' care plans, is essential for comprehensively assessing the effectiveness of the AN‐ACC in facilitating the delivery of allied health care.

## CONFLICT OF INTEREST STATEMENT

No conflicts of interest declared.

## Data Availability

The data that support the findings of this study are available on request from the corresponding author. The data are not publicly available due to privacy or ethical restrictions.
